# The Importance of Socioeconomic Factors Associated with Maternal Nutrition Knowledge and Undernutrition Among Children Under Five

**DOI:** 10.3390/nu17213355

**Published:** 2025-10-24

**Authors:** Arie Dwi Alristina, Rizky Dzariyani Laili, Éva Nagy, Helga Judit Feith

**Affiliations:** 1 Health Sciences Division, Doctoral College, Semmelweis University, 1085 Budapest, Hungary; alristina.arie@phd.semmelweis.hu; 2 Nutrition Department, Sekolah Tinggi Ilmu Kesehatan Hang Tuah Surabaya, Surabaya 60244, Indonesia; rizkylaili@stikeshangtuah-sby.ac.id; 3 Department of Social Sciences, Faculty of Health Sciences, Semmelweis University, 1085 Budapest, Hungary; nagy.eva@semmelweis.hu

**Keywords:** socioeconomic, maternal nutrition knowledge, stunting, underweight, wasting, mother

## Abstract

**Background**: Socioeconomic factors may influence maternal nutrition knowledge (MNK), which directly affects the nutritional status of children under five. This study aims to explore the importance of socioeconomic factors associated with MNK and nutritional status. **Methods**: This cross-sectional study focused on mothers of children aged 36–59 months (n = 657). A structured questionnaire was employed to collect data on socioeconomic factors. Anthropometric measurements were taken to assess nutritional status. The Boruta algorithm, implemented using R Studio version R.4.5.1, was used to identify the most important socioeconomic factors associated with MNK and nutrition status. **Results**: The analysis revealed that socioeconomic status (SES) emerged as an important factor associated with MNK and nutrition status, particularly stunting and wasting. However, SES was not confirmed as an important factor associated with underweight. This study uncovered a bidirectional relationship between child nutrition outcomes; underweight was found to be an important factor related to stunting and wasting, whereas stunting and wasting were important factors for underweight. Furthermore, infant and young child feeding (IYCF) indicators, such as weaning practices and exclusive breastfeeding (BF), were found to be important factors for stunting and wasting. **Conclusions**: The interlinkage among forms of undernutrition, where each nutritional outcome is related to other outcomes, underscores the importance of comprehensively addressing child undernutrition, rather than focusing on single outcomes independently. Moreover, the association between SES and MNK, wasting, and stunting supports approaches based on holistic and multi-sectoral strategies to reduce poverty by WASH programs, promote IYCF practices, and improve healthcare access by providing health insurance coverage.

## 1. Introduction

Malnutrition is a prevailing global health concern, especially for children under five. In 2023, UNICEF, WHO, and the World Bank Group highlighted that wasting and stunting remain significant challenges to child survival and development. Globally, approximately 45 million children worldwide suffer from wasting, a severe form of acute malnutrition. Stunting, on the other hand, affects approximately 148.1 million (22.3%) children. Although its prevalence is decreasing, it reflects chronic undernutrition that leads to limited physical growth and cognitive development and puts children at a lifelong disadvantage. In contrast, wasting is a more immediate threat to survival, and prompt detection and treatment are necessary, given that the majority of cases occur in Asia and Africa [1]. Child survival, long-term health, and socioeconomic progress are threatened by these forms of malnutrition, which lead to lifelong disadvantage. Adults who experienced stunting in childhood tend to earn a lower income in adulthood [1,2].

Undernutrition is primarily manifested as stunting (representing chronic undernutrition) and wasting (representing acute undernutrition), whereas underweight may result from either stunting, wasting, or a combination of both conditions. Undernutrition adversely affects children under five, as they require adequate nutrition to support rapid physical growth and cognitive development during early childhood. The Food and Nutrition Technical Assistance III (FANTA) stated that adequate nutrition is essential for a child’s optimal development, especially during the first 1000 days of life (from conception to the child’s second birthday), a crucial phase marked by acceleration in growth rate when deficiencies can have lifelong impacts [3]. The symptoms of malnutrition are particularly apparent in stunting (height-for-age Z score (HAZ) below −2 standard deviations) and wasting (weight-for-height Z score (WHZ) below −2 standard deviations), which are indicative of chronic and acute undernutrition, respectively [4,5]. In 2024, approximately 15.2% of children were underweight globally [6], reflecting low weight-for-age that may result from either stunting, wasting, or both conditions.

The burden of malnutrition is a challenge in developing countries as a consequence of poverty and limited access to nutrient-rich foods [7]. Southeast Asia is experiencing the triple burden of malnutrition [2]: stunting, micronutrient deficiencies (“hidden hunger”), and susceptibility to obesity, which challenge sustainable health development. Indonesia is not an exception, as undernutrition remains a public health concern despite its remarkable economic growth. In terms of national prevalence, there was a decline in stunting from 27.7% in 2019 to 21.6% in 2022; however, rates remain above the WHO threshold of 20% [8]. A study found that, in 2022, 6.8% of children under five were wasted and 2.1% were severely wasted [1]. Urban areas, such as Surabaya, Indonesia, are particularly vulnerable due to persistent socioeconomic disparities.

Maternal nutrition knowledge (MNK) is underscored as a critical factor for child nutritional status [9,10]. Numerous studies have demonstrated that providing effective nutrition education improves mothers’ knowledge, which in turn affects child feeding practices and reduces the risk of malnutrition in children. For instance, a study conducted in Kupang Regency, Indonesia, showed that family-centred education using media such as booklets and food samples was effective in improving nutritional practices among pregnant women, which in turn led to reduced stunting [11]. Various interventions have been implemented to enhance MNK and child nutritional status, including nutrition education [12,13], brainstorming [14], and audiovisual demonstrations [15]. Nutritional health education can be delivered in booklets [16], guidebooks, leaflets, and internet technology applications (ICT or webApps or APK) [9,15,17]. These methods have demonstrated significant improvements in MNK, showing its relevance to positive child health outcomes, including reduced prevalence of stunting [10]. However, the effectiveness of such interventions requires that mothers have the opportunity and ability to acquire nutrition knowledge, which household resources and socioeconomic conditions may influence. A mother’s ability to access, comprehend, and utilise educational resources is not uniform. Also, it is profoundly mediated by their household’s socioeconomic status (SES), which governs exposure to information, formal education, and technology [18]. However, research to identify socioeconomic factors that impact MNK is still limited.

MNK and feeding practices are two closely related but different concepts. MNK is conceptualised as a mother’s knowledge of nutrition and dietary requirements, food options, and child feeding principles [19]. In contrast, feeding practices refer to the actual behaviours used in daily feeding, including exclusive breastfeeding, meal frequency, and dietary diversity [20]. Previous findings revealed that good nutrition knowledge does not always translate into appropriate feeding practices [21,22]. Therefore, the distinction between MNK and feeding practices should be elaborated to identify socioeconomic factors related to both mothers’ knowledge and their practice in supporting adequate child nutrition. 

According to the WHO’s Conceptual Framework for Action on the Social Determinants of Health (CSDH) and UNICEF’s modified framework, we conceptualise socioeconomics as structural factors that may associate with MNK and child nutritional status. More precisely, it comprises the broader socioeconomic factors that relate to women’s resources through education, occupation, and income [23,24]. These conditions affect intermediate factors of material circumstances (food accessibility related to the affordability of food, living, and working conditions) and behavioural factors, among which MNK is one key behavioural resource that shapes feeding, caregiving, and the use of health services. Therefore, this study aims to identify the important socioeconomic factors related to MNK and the nutritional status of children under five in Surabaya, Indonesia. These findings, derived from the Boruta analysis, may guide policymakers in prioritising factors for implementing a comprehensive public health intervention.

## 2. Materials and Methods

### 2.1. Study Design

A cross-sectional design was used for the current study. A multi-stage cluster random sampling method was employed, using census blocks provided by the Indonesian Bureau of Statistics. This process involved randomly selecting two sub-districts from each district. The sample size for each district was determined proportionally, based on the number of children under five in each district relative to the overall population of children under five in all selected districts. The study targeted mothers with children aged 36 to 59 months, ultimately including 657 households in the analysis. When a household had more than one eligible child, only one child was considered to participate in the analysis. Before participation, literate participants provided written consent, while for those who were illiterate, consent was documented with a thumbprint in the presence of a witness. The confidentiality of the participants was strictly protected throughout the study to ensure their anonymity and privacy [25].

### 2.2. Data Collection

Data collection was carried out through face-to-face interviews using a structured questionnaire, scheduled to coincide with routine child monitoring sessions in each sub-district. Mothers with children aged 36–59 months registered at the Public Health Centre were eligible to participate in the study. Mothers or children with a serious illness, disabilities affecting anthropometric measurement, and incomplete data were excluded. Interviews took between 30 and 45 min per participant. Procedures were conducted in public health centres to create an environment that was both comfortable and familiar. The interviews were administered by trained enumerators who recorded the anthropometrics. All enumerators were given further training in the administration of questionnaires, interview ethics, and standardised methods for recording data before data collection.

Independent variables, including sociodemographic, socioeconomic, and related information, were collected using a structured questionnaire. Sociodemographic factors included child gender, family size, siblings under five, and child health insurance. Family size was defined as the number of people in the nuclear family (parents and children). SES is a measure of where an individual stands within society and typically includes assessing income, level of education, occupation, and subjective wealth status, which refers to financial prosperity (assets, such as house and car ownership) [26,27,28]. In this study, SES was computed based on family income, mother’s education, mother’s employment, family ownership (car and house), and subjective wealth status. SES levels were measured by total scores in tertiles and classified as low SES (≤33.33%), middle SES (33.34–66.67%), and high SES (>66.67%) [29]. Mother’s employment was categorised into two groups: employed (formal or informal), which included working in the past 12 months, regardless of the type of job (full-time or part-time), and unemployed, representing mothers not involved in any income-generating activity. Quality of life was measured using a combination of objective indicators (such as family income, household equipment, education level, and employment status of mother) and subjective wealth status (self-reported wealth status of respondent). Subjective wealth status was measured using a single self-report question that asked respondents to compare their wealth status with that of other families in their community, using three response options: (1) rather better off, (2) average, and (3) rather worse off [30,31,32].

Breastfeeding (BF) practices were assessed through maternal recall, including the time BF commenced, the condition of BF, and the period for which the mother had applied IYCF indicators based on WHO and UNICEF guidelines to the index child [33]. BF practices were measured using a structured questionnaire according to IYCF guidelines provided by WHO, a validated tool designed to evaluate feeding behaviours, such as exclusive BF, initiation BF, and duration. Because the data were based on maternal recall, recall bias could not be excluded entirely.

The dependent variables were identified as MNK and child nutrition status. MNK can be defined as the understanding of nutrients, dietary practices, and food preferences by a mother that helps maintain health and provide proper growth and development for the infant. We employed the Qualtrics Survey General Nutrition Knowledge Questionnaire—Revised (GNKQ-R), which includes eighteen questions with a total score of 52, to assess mothers’ general nutrition knowledge. The GNKQ-R is considered a reliable instrument for evaluating nutritional knowledge in the adult population and is not specifically designed to measure knowledge related to child nutrition [34]. We divided MNK into three levels by using the tertile calculations as cut-off points [35]. According to MNK scores, we classified individuals as follows: low MNK (score ≤ 17), moderate MNK (score 18–34), and high MNK (score ≥ 35). While child nutrition status indicators were measured, the indicators included the weights and heights of the children. Weight was measured using a calibrated digital scale (SECA) and zeroed before each measurement. Children were weighed with minimal clothing and without footwear and asked to remain as still as possible. The heights of the children were measured with them standing upright and barefoot. All measurements were conducted in duplicate by 12 trained enumerators who received standardised training on anthropometric techniques before data collection. Supervisors conducted random spot checks to ensure adherence to protocols, and daily calibration of equipment was performed. Inter-observer variation was minimised through repeated training and pilot testing before the survey [6].

### 2.3. Data Analysis

Nutritional status was calculated using the WHO Child Growth Standards [6], with classifications generated through the WHO ANTHRO software version 3.2.2. The collected data were converted into Z scores for key indicators: HAZ, WHZ, and WAZ. Stunting, wasting, and underweight were defined as HAZ, WHZ, and WAZ values below −2 standard deviations from the median for the reference population, respectively [6].

Demographic and household characteristics were analysed as categorical variables to interpret frequency distributions using IBM SPSS version 27.0. Further statistical analysis was performed with the Boruta package using R studio version R.4.5.1, which was used in previous research to identify the most important factor for dependent variables [36]. This process enhances machine learning model performance by eliminating irrelevant variables, thereby simplifying models, improving speed, and increasing accuracy [37]. It is available via the Comprehensive R Archive Network (CRAN) in the Boruta Package. The Boruta algorithm provides strong all-relevant feature selection by identifying every factor that is statistically significant for the outcome. The Boruta feature selection algorithm was employed to identify factors such as MNK and undernutrition outcomes, including stunting, wasting, and underweight. The Boruta algorithm helps confirm relevant features and discard irrelevant ones, making it resistant to noise and overfitting in random forest models. We prefer Boruta factors comprehensively. This non-parametric approach is important in nutritional science studies and in assessing non-linear relationships, unlike parametric methods in SPSS, which rely on parametric assumptions and stepwise methods. In contrast, parametric methods in SPSS rely on parametric assumptions and stepwise methods, which can miss important correlated features or those with complex interactions. Importance scores for these variables were calculated and visualised through boxplots. In these visualisations, blue boxplots represent the minimum, average, and maximum Z scores of the shadow attributes for dependent variables; red boxplots represent rejected features; yellow boxplots represent tentative variables; and green boxplots represent confirmed important variables.

## 3. Results

### 3.1. Study Population

This study included 657 mothers with children aged between 36 and 59 months, 49.6% of whom were boys and 50.4% of whom were girls. Among the participants, 25.3% of the children were reported to be stunted, while 16.1% and 22.5% were classified as wasted and underweight, respectively. Undernutrition clustered with underweight: 51.4% were also wasted, and 55.4% were stunted. Interestingly, 16.2% of the children bore the triple burden of being underweight, stunted, and wasted. Among normal-weight children, wasting was observed in 5.9% and stunting in 16.5%, while overlap between stunting and wasting was observed in 3.7% (Table S1).

SES was determined by family income, mother’s education, mother’s employment, car ownership, house ownership, and subjective wealth status. This research revealed that the majority of families (39.4%) had an income below the minimum wage per month in 2024, which was IDR 4,725,479 (approximately EUR 272) [38]. Monthly income was below IDR 5,700,000 (approximately EUR 328) in as much as 44.3% of families. The data also showed that most mothers had a high school education (57.2%) and were unemployed (57.7%). Furthermore, families mostly had no house ownership (67.6%) and no car ownership (87.4%). Most mothers (66.4%) reported that their family had an average subjective wealth status, and they evaluated their overall situation as neither particularly good nor poor (Table 1).

The SES of the households was categorised into three levels: high, middle, and low SES. The findings revealed that only 5.0% of the households were classified as high SES, indicating a small proportion of families with significant economic resources. The majority of respondents (89.5%) fell into the middle SES category, reflecting households with average economic stability. In comparison, 5.5% were identified as low SES, highlighting the population facing a lack of financial resources to attain a decent standard of living. MNK was measured and specified in three levels: low, moderate, and high. Even though most of the respondents demonstrated a moderate level of MNK (72%), we still found that 27.4% of them had low MNK and fewer mothers had high MNK (0.6%).

Children who indicated undernutrition experienced stunting (25.3%), wasting (16.1%), and underweight (22.5%). Furthermore, child feeding indicators demonstrated suboptimal exclusive BF, with only 37% of children being exclusively breastfed. In contrast, the percentage of children who had ever been breastfed was 91.9%. Also, a low rate of proper initiation BF was found, with 32.6% experiencing initiation BF within 1 h after giving birth (Table 1).

### 3.2. The Important Factors for MNK

**Figure 1:**
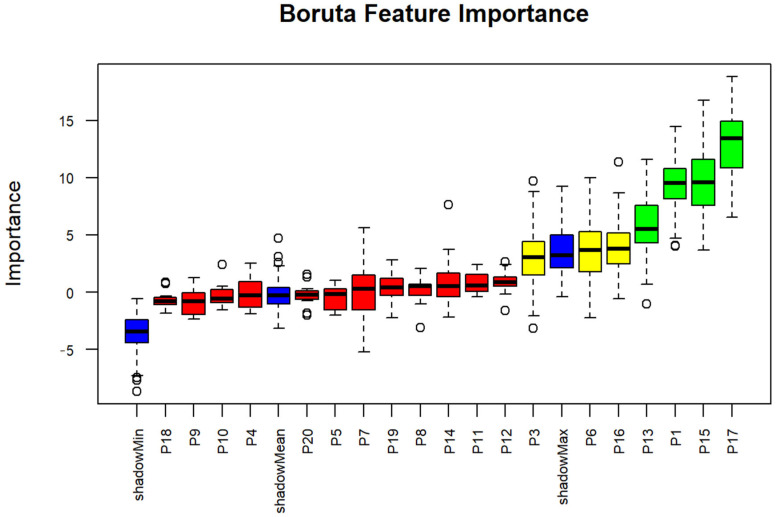
Boxplots derived from Boruta algorithm for MNK. The circles indicate outlier values, iterations in which the computed feature importance was much higher or lower than others for that same feature. Blue boxplots represent the minimum, average, and maximum Z scores for a shadow attribute. Z scores for attributes that were rejected, tentative, or confirmed are depicted by the red, yellow, and green boxplots, respectively.

Importance scores indicate the relative importance of each variable in influencing the outcome, as determined by the Boruta feature selection algorithm. Higher values indicate a closer relation to the outcome variables. The Boruta analysis was used to determine the most important factor influencing MNK. The results highlighted several critical findings and may reveal important factors in influencing MNK and child nutrition. SES and initiation BF scores emerged as the most important factors for MNK, respectively. Furthermore, stunting and child gender were indicated as tentative factors for MNK, suggesting their moderate association with MNK. Furthermore, SES variables found to be important in influencing MNK were mother’s education and family income (Table 2, Figure 1).

### 3.3. The Importance Factors for Stunting

**Figure 2:**
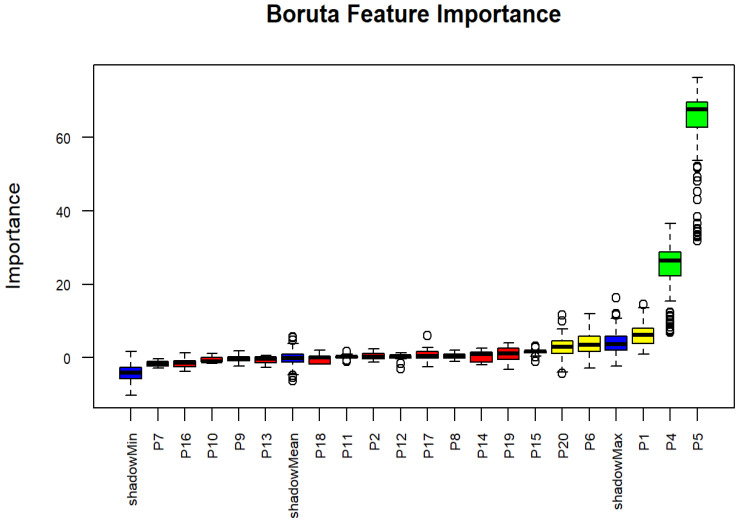
Boxplots derived from Boruta algorithm for stunting status. The circles indicate outlier values, iterations in which the computed feature importance was much higher or lower than others for that same feature. Blue boxplots represent the minimum, average, and maximum Z scores for a shadow attribute. Z scores for attributes that were rejected, tentative, or confirmed are depicted by the red, yellow, and green boxplots, respectively.

Wasting and underweight were identified as important factors, contributing significantly to the model’s ability to accurately influence stunting status, and were confirmed by Boruta analysis. Additionally, SES and child gender were revealed to exert moderate effects on stunting. The SES indicator of perceived quality of life was also identified as a moderate importance factor for stunting, as shown in Table 3 and Figure 2.

### 3.4. The Importance Factors for Wasting

**Figure 3:**
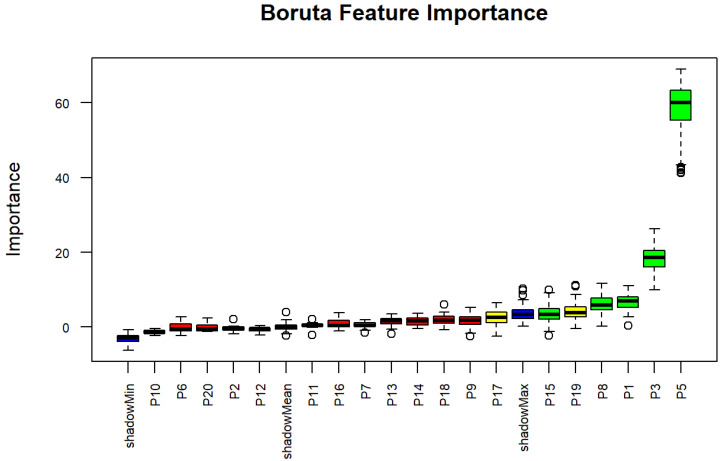
Boxplots derived from Boruta algorithm for wasting status. The circles indicate outlier values, iterations in which the computed feature importance was much higher or lower than others for that same feature. Blue boxplots represent the minimum, average, and maximum Z scores for a shadow attribute. Z scores for attributes that were rejected, tentative, or confirmed are depicted by the red, yellow, and green boxplots, respectively.

The study also sought to identify key factors for predicting wasting status using the Boruta algorithm. The analysis found several socioeconomic factors to be important factors associated with wasting. SES was revealed as an important factor for wasting, following stunting, underweight, and mother’s age, which also emerged as highly significant factors, strongly influencing wasting status. In addition, mother’s education was also an important factor for wasting. Moderately important factors, such as family income and house ownership, were also identified. Several factors were deemed non-significant in predicting wasting status, including MNK, child gender, child health, under-five siblings, exclusive BF, and ever BF. These findings, presented in Table 4 and Figure 3, provide valuable insights into the critical and non-critical factors correlated with wasting status in children under five.

### 3.5. The Importance Factors for Underweight

**Figure 4:**
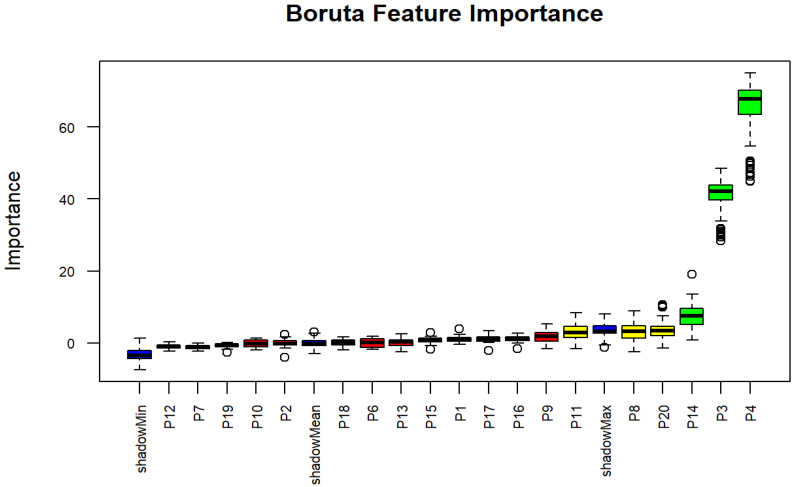
Boxplots derived from Boruta algorithm for underweight status. The circles indicate outlier values, iterations in which the computed feature importance was much higher or lower than others for that same feature. Blue boxplots represent the minimum, average, and maximum Z scores for a shadow attribute. Z scores for attributes that were rejected, tentative, or confirmed are depicted by the red, yellow, and green boxplots, respectively.

SES was not confirmed as an important factor for underweight, even though subjective wealth status was revealed as a moderately important factor. In contrast, stunting, wasting, and weaning practices, which were classified as important factors by Boruta analysis, contributed significantly to the model to predict underweight status. Additionally, moderate factors were found, including mother’s age and exclusive BF, as illustrated in Table 5 and Figure 4. These findings offer critical insights into the factors for underweight status and may contribute to health priority programs and modelling initiatives, as well as related studies.

## 4. Discussion

The current study has underscored the importance of socioeconomic factors, including family income, maternal education, maternal employment, car ownership, house ownership, and subjective wealth status, as important factors associated with MNK and nutritional status in children aged 36–59 months. Employing the Boruta algorithm, SES was identified as the most important factor across models for MNK and undernutrition status, particularly wasting. SES was also confirmed to be an important factor associated with stunting. The results provide evidence that child undernutrition is a multidimensional problem, where socioeconomic factors are linked to mothers’ ability to access and understand nutrition knowledge. These factors are also connected to their capacity to utilise their available resources, such as household income, healthcare, and nutritious foods, to support optimal child growth.

### 4.1. SES and MNK

This study found that MNK has a strong association with SES and early initiation of BF. A previous study highlighted that higher SES is an important driver for exposure to health information; uptake of antenatal and postnatal services also influences engagement with healthcare services [39,40,41]. Mothers from low-SES backgrounds may additionally face barriers, as limited reading literacy is a prerequisite for obtaining health information. These barriers can hinder their ability to obtain and interpret written health information. Furthermore, these challenges, combined with difficulties in utilising healthcare services and competing economic priorities, may reduce their capacity to support optimal child nutrition. Recent findings indicated that maternal knowledge mediates the association between SES and feeding practices, leading to better dietary diversity and growth among children [42,43]. In this study, SES classified by education, employment, income, and wealth condition aligns with previous studies that highlight the relationship between education, income, and occupation and MNK levels [41].

Interestingly, maternal education, as one of the SES factors, was revealed as an important factor for MNK. This study aligns with previous findings, which highlighted an important relationship between maternal education level and MNK [44,45]. A previous study analysed the relationship between mother’s education and the level of exposure and access to media. The effect of a mother’s higher level of education can be partly explained by their access to information, through newspapers, television, social media, posters, and/or other internet and communication technologies, in general and with respect to child health and nutrition [46,47]. Moreover, their ability to express and think autonomously based on acquired knowledge, as well as the effects of their immediate environment and community, also helps explain how they make choices about nutrient healthcare for their children.

A recent study revealed that family income was a predictor for nutrition knowledge among pregnant women [48]. This finding was similarly made in the current study, which indicates that family income is an important factor for MNK. Family income can influence access to various resources that contribute to nutrition knowledge. For instance, it can determine a family’s ability to acquire diverse and nutrient food preferences, which can, in turn, familiarise mothers with a broader spectrum of food groups and their nutritional significance. Additionally, family income may influence access to health and nutrition information, such as books, magazines, or online resources focused on child health and nutrition. It can also affect the ability to seek out and utilise healthcare services that provide dietary counseling, whether from general practitioners, nurses, or specialised nutritionists. These various avenues, influenced by a family’s financial capacity, collectively contribute to a pregnant woman’s acquisition of comprehensive nutrition knowledge, enabling her to make informed decisions regarding her and her child’s dietary well-being.

Additionally, stunting status was also identified as an important factor for MNK in the Boruta model, which may reflect a bidirectional relationship. While mothers whose child is underweight may actively seek nutrition information once growth faltering has been diagnosed, limited pre-diagnosis knowledge might lead to poor feeding practices and, consequently, an increased risk of underweight. These results are inconsistent with previous research that did not find an association between MNK and malnutrition [49,50]. It may be that improved MNK is associated with having experienced malnutrition in children. Consequently, mothers find out more about child feeding and healthy food for their children.

### 4.2. SES and Child Undernutrition

The association of SES with wasting and stunting and its indirect association with underweight suggest that SES is an important factor associated with multiple manifestations of undernutrition among children. This is consistent with previous studies that reported that children in lower-SES households are more vulnerable to stunting. Children in low- and middle-income countries from the poorest wealth quintiles are more likely to be stunted, wasted, or underweight compared to those in the highest quintiles [51]. SES was the most important factor for stunting and wasting, consistent with recent studies that have shown positive relationships between higher household wealth and improved child growth outcomes [52,53]. In terms of mechanism, SES works in different ways on child nutrition by affecting household food security [54] and dietary diversity [43], enabling access to good healthcare facilities, better sanitation, and hygiene conditions [55]; it is also indirectly related to maternal education, which in turn is linked to improved feeding practices [56], and better job opportunities, which may enhance household food security [25]. Importantly, mothers being in employment does not necessarily imply a detriment to child nutrition, as children of working mothers also have access to care and feeding from other caregivers (fathers, grandmothers, or others) who can provide sufficient or poor-quality care. Previous research has demonstrated that father and other family member involvement in child feeding and care can mitigate the early adverse nutrition effects of maternal employment [57,58].

The present study revealed that SES is a moderately important factor for stunting, which is related to subjective wealth status in households. It aligns with previous research that found the relationship between SES and a household’s ability to secure a diverse, nutrient-rich diet and that SES constraints may limit access to maternal healthcare [43,59], safe water, and sanitation facilities [55]. Previous research suggested that lower SES also correlates with lower maternal education levels [41,60]. Aligned with this study, it also confirmed that SES is one important factor for MNK, which in turn reduces the likelihood of adopting optimal IYCF practices.

This study highlights that SES has an important association with wasting among children and may increase vulnerability to acute nutritional deprivation. Wasting has been widely documented as a risk factor for increased vulnerability related to the immune system and risk of infection [61]. Furthermore, consistent with previous research, households that have lower purchasing power often rely on the worst alternative foods, such as substitutes for cereal-based processed foods [62], and lack access to healthcare during illness episodes, which contribute to accelerated weight loss and wasting [63,64]. Similar to what has been reported in Sub-Saharan African countries and South Asia, low household income was consistently associated with increased odds of wasting [65,66], particularly in the context of high infection burdens [65].

Furthermore, this study emphasises that the mother’s education is an important factor associated with child nutrition status, particularly wasting and stunting. It may provide the knowledge required to feed and medicate children optimally, including information about various nutrients and recognising illness symptoms, regardless of financial constraints. Parallel with this, a previous study revealed that improving maternal education is essential to address child undernutrition (stunting, wasting, and underweight), as it provides mothers with the necessary knowledge input for better childcare and nutrition practices [67].

For underweight children, the pathway of effect of SES is indirect through stunting and wasting, since stunting and wasting are important factors for underweight. Likewise, stunting and wasting were identified as important factors for underweight, indicating a strong interrelationship among these indicators of child malnutrition. However, a previous study found that low-SES households have high cumulative risks because the same underlying socioeconomic barriers contribute to both acute and chronic forms of malnutrition, such as poor dietary quality, inadequate feeding frequency, and recurrent infections, which directly lead to underweight [68]. Moreover, the present study indicated that IYCF indicators, particularly weaning practices nd exclusive BF, were important factors for underweight, suggesting a possible link with undernutrition status, as studied in prior studies [69,70].

In largely homogenous low-socioeconomic status areas, however, SES may have less of an effect on undernutrition. Instead, feeding culture (i.e., traditional feeding practices and household food access) and MNK appear to have a greater impact on maternal and children’s dietary patterns [71] than maternal education [71]. Mothers in Northern Vietnam strive to feed their preschool children with safe and healthy food that caters to their taste so that the children eat more [72]. Their feeding behaviours are determined by maternal decision-making in food choices, which is shaped by family eating habits and the mixed food environment in which healthy and unhealthy foods are available. Nutritional intervention must, therefore, be implemented at multiple levels of society, not in an isolated manner, as addressing malnutrition is challenging. A multifaceted approach, which certainly includes revamping MNK to encourage healthier dietary patterns and thus dietary diversity, can lead families towards a better nutritional status.

Previous studies also indicated that differences in SES may be less significant compared to other factors, such as cultural feeding norms (food taboos and food prohibitions) and MNK, in determining dietary patterns for mothers and children [73,74,75]. This highlights the importance of a multi-level analysis that takes into consideration not only overall economic inequality, but also localised socio-cultural factors. Consequently, interventions need to focus on culture, integrating SES improvements and tailored education to reduce cultural barriers and improve MNK.

These studies underscore the importance of IYCF indicators in child growth and nutritional status development. Weaning practices are highlighted as an important factor in underweight, consistent with earlier findings that indicate a significant relationship between weaning practices and underweight [69]. In addition, exclusive BF was revealed to be a less definitively important factor for underweight than weaning practices, suggesting that while exclusive BF may influence nutritional status changes over time, its effect needs further investigation. Considering that MNK was not found to be associated with child nutrition outcomes, this study suggests that MNK may not always lead to appropriate practices, such as in IYCF behaviours. Poverty, family decision-making, and cultural feeding practices can be barriers to applying knowledge in practice. This knowledge gap highlights a crucial need for further research and the development of program designs.

During the study period, several maternal and child nutrition programs were also underway in Indonesia. These comprised community-level services through Posyandu, such as promoting BF and complementary feeding, as well as government programs like Program Keluarga Harapan (PKH), which offered conditional cash transfers. Community health workers were also involved in disseminating nutrition information, promoting child feeding, and conducting mass media efforts on a national scale to prevent stunting. However, the scale and intensity of these interventions varied between regions, and mothers with older children might have experienced contrasting program exposure due to policy changes over time. Therefore, some mothers may have experienced recent increases in knowledge concerning nutrition (as a result of these programmes). Still, they had not yet fully reflected this new knowledge in the form of optimal child feeding practices or nutritional status. In this context, our findings underscore the need to further reinforce economic and educational interventions (such as poverty reduction, women’s economic empowerment, and culturally adapted antenatal education) and to continue targeting socioeconomic factors related to MNK, stunting, wasting, and underweight.

### 4.3. Strengths and Limitations

This study combines a more detailed assessment using the Boruta algorithm for feature selection, which improves methodological rigour by enabling an objective recapitulation of the most important factor and reducing bias compared to regression models. Boruta uses shadow features and selects the only relevant models to target the variable based on the random forest importance measure. Moreover, this study integrates maternal, child, and household factors from various settings into distinct domains (i.e., SES, MNK, and child nutritional status), allowing for a more comprehensive description of the multi-level structural factors alongside the indicators of IYCF practices. Although our results are associations and not direct causal pathways, they suggest the relevance of integrated, multi-sectoral approaches to child nutrition [76,77]. However, some limitations should be considered. First, the Boruta algorithm prohibits causal inference, while observed associations, particularly for some factors such as MNK and child nutritional status, may reflect reverse causality. Self-reported measures of BF practices (including initiation BF, exclusive BF, ever BF, and weaning practices) and maternal knowledge have the potential for recall and social desirability bias, which could result in non-differential misclassification. Secondly, the GNKQ-R is not specifically designed for IYCF practices; however, it was applied in this study as a proxy measure of maternal knowledge on nutrition, and this limitation is acknowledged. Thirdly, the research instrument (questionnaire) contained only closed-ended questions. By implication, this meant that participants had limited response options. When SES was not found to be an important factor, we could not determine which other variable may directly influence the dependent variable. A qualitative study was suggested for further research to explore uncovered information. Lastly, Boruta could be supplemented by other statistical tests (e.g., multiple linear regression) to investigate the trend of association of SES levels and maternal nutrition knowledge (MNK) with undernutrition outcomes after adjusting for covariates.

Furthermore, this study indicates that decision-makers, policymakers, and program implementers should promote integrated approaches to tackle both socioeconomic constraints and nutrition-specific practices, such as combining social protection schemes with improving MNK or targeted nutrition counselling to improve child growth outcomes. Further intervention studies utilising longitudinal and causal designs are required to validate pathways, test the efficacy of bundled interventions, and examine inherent and modifiable factors, such as cultural infant feeding practices, that may impact child health and nutrition potential.

## 5. Conclusions

**Figure 5:**
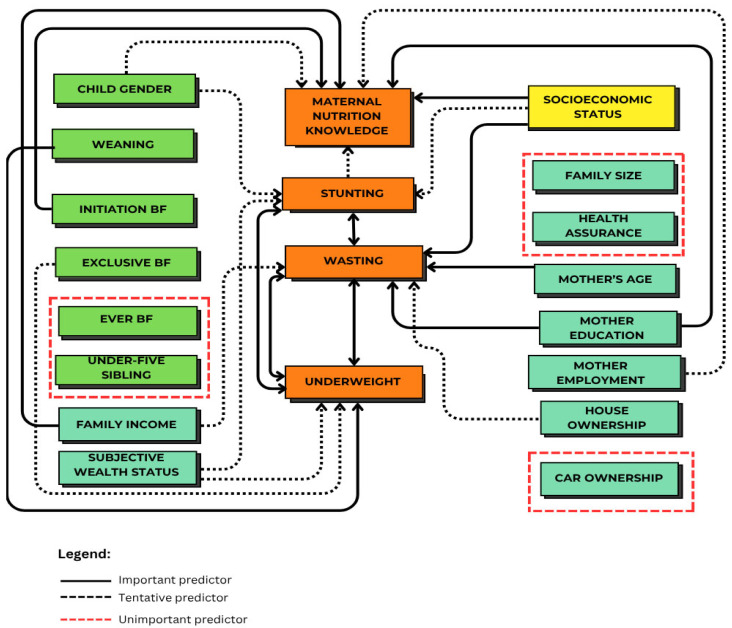
The importance of socioeconomic models for MNK and undernutrition.

The importance of socioeconomic factors for MNK and child undernutrition outcomes is underscored, as shown in Figure 5. SES was the most important factor for MNK and stunting, suggesting that interventions addressing both economic constraints and nutritional education may be required to enhance the impact of nutrition programs. Socioeconomic factors, such as family income and the mother’s education, are the most important factors influencing MNK. At the same time, child feeding indicators (weaning practices and exclusive BF) are important factors for undernutrition, as well as initiation BF related to MNK, especially for those whose long-term child growth outcomes are not severely affected by structural SES. These findings highlight the need to simultaneously address socioeconomic factors and MNK for programmatic and policy action in reducing child undernutrition. In addition, these findings support a multi-sectoral approach, including economic, maternal education, and nutrition-specific interventions, to break the intergenerational cycle of malnutrition and enable sustainable improvements in early child health and development in resource-limited settings. Interventions aimed at enhancing SES, strengthening access to health services, and targeted nutrition education are required in tackling the root causes of child undernutrition.

## Figures and Tables

**Table 1:** Characteristics of the study participants.

Variables	Frequency	Percentage (%)
SES:		
Low	36	5.5
Middle	588	89.5
High	33	5.0
MNK:		
Low	180	27.4
Moderate	473	72.0
High	4	0.6
Mother’s age:		
<20 years	1	2.0
20 to 29 years	100	15.2
30 to 39 years	336	51.1
≥40 years	220	33.5
Family size:		
≤3 members	114	17.4
4–6 members	449	68.3
>6 members	94	14.3
Under-five siblings:		
≤2 children	621	94.5
≥3 children	36	5.5
Child gender:		
Boy	326	49.6
Girl	331	50.4
Child health insurance:		
Yes	353	53.7
No	304	46.3
Stunting:		
Yes	166	25.3
No	491	74.7
Wasting:		
Yes	106	16.1
No	551	83.9
Underweight:		
Yes	148	22.5
No	509	77.5
Ever BF:		
Yes	604	91.9
No	53	8.1
Exclusive BF:		
Yes	245	37.3
No	412	62.7
Initiation BF:		
Within 1 h	214	32.6
After 1 h or more	443	67.4
Weaning practices:		
Less than 6 months	124	18.9
Between 6 months and 24 months	330	50.2
24 months or more	203	30.9
Mother’s education:		
No education	4	0.6
Primary school	179	27.2
High school	376	57.2
Higher degree and above	98	14.9
Mother’s employment status:		
Employed	278	42.3
Unemployed	379	57.7
Family income:		
≤IDR 2,300,000 per month	259	39.4
IDR 2,300,001–IDR 4,500,000 per month	291	44.3
IDR 4,500,001–IDR 5,700,000 per month	61	9.3
IDR 5,700,001–IDR 7,000,000 per month	26	4.0
IDR 7,000,001–IDR 10,000,000 per month	13	2.0
>IDR 10,000,001 per month	7	1.1
Car ownership:		
Yes	83	12.6
No	574	87.4
House ownership:		
Yes	213	32.4
No	444	67.6
Subjective wealth status:		
Rather better off	202	30.7
Average	436	66.4
Rather worse off	19	2.9

**Table 2:** Factors’ importance scores derived from Boruta algorithm for MNK.

Code Variables	Mean Imp.	Median Imp.	Min. Imp.	Max. Imp.	Norm Hits	Decision
P1. SES	9.398	9.543	4.025	14.438	0.980	Confirmed
P3. Stunting	3.003	3.024	−3.153	9.706	0.374	Tentative
P4. Wasting	−0.135	−0.301	−1.896	2.507	0.000	Rejected
P5. Underweight	−0.505	−0.182	−2.009	1.029	0.000	Rejected
P6. Child gender	3.812	3.649	−2.207	9.991	0.475	Tentative
P7. Health insurance	0.202	0.276	−5.197	5.626	0.051	Rejected
P8. Mother’s age	0.064	0.518	−3.104	2.075	0.000	Rejected
P9. Family size	−0.901	−0.789	−2.344	1.277	0.000	Rejected
P10. Under-five siblings	−0.243	−0.551	−1.527	2.406	0.000	Rejected
P11. Exclusive BF	0.837	0.589	−0.403	2.412	0.000	Rejected
P12. Ever BF	0.887	0.890	−1.600	2.638	0.000	Rejected
P13. Initiation BF	5.892	5.516	−1.014	11.589	0.727	Confirmed
P14. Weaning practices	0.938	0.550	−2.183	7.634	0.020	Rejected
P15. Mother education	9.521	9.556	3.691	16.766	0.980	Confirmed
P16. Mother employment	3.806	3.795	−0.569	11.37	0.556	Tentative
P17. Family income	13.013	13.43	6.525	18.811	1.000	Confirmed
P18. Car ownership	−0.636	−0.815	−1.824	0.857	0.000	Rejected
P19. House ownership	0.313	0.421	−2.235	2.812	0.000	Rejected
P20. Subjective wealth status	−0.225	−0.202	−2.006	1.542	0.000	Rejected

**Table 3:** Factors’ importance scores derived from Boruta algorithm for stunting status.

Code Variables	Mean Imp.	Median Imp.	Min. Imp.	Max. Imp.	Norm Hits	Decision
P1. SES	6.075	6.164	0.851	14.483	0.657	Tentative
P2. MNK	0.301	0.181	−1.263	2.455	0.000	Rejected
P4. Wasting	24.503	26.458	6.830	36.536	1.000	Confirmed
P5. Underweight	62.803	67.701	31.805	76.340	1.000	Confirmed
P6. Child gender	3.912	3.412	−2.931	12.018	0.465	Tentative
P7. Health insurance	−1.720	−1.764	−2.786	−0.243	0.000	Rejected
P8. Mother’s age	0.388	0.618	−1.079	1.977	0.000	Rejected
P9. Family size	−0.382	−0.413	−2.300	1.840	0.000	Rejected
P10. Under-five siblings	−0.475	−1.004	−1.650	1.117	0.000	Rejected
P11. Exclusive BF	0.198	0.179	−1.073	1.593	0.000	Rejected
P12. Ever BF	−0.052	0.467	−3.014	1.290	0.000	Rejected
P13. Initiation BF	−0.645	−0.272	−2.602	0.507	0.000	Rejected
P14. Weaning practices	0.387	0.918	−1.929	2.606	0.010	Rejected
P15. Mother education	1.549	1.685	−1.023	3.082	0.010	Rejected
P16. Mother employment	−1.570	−1.425	−3.790	1.211	0.000	Rejected
P17. Family income	0.708	0.473	−2.582	6.074	0.020	Rejected
P18. Car ownership	−0.356	−0.050	−1.767	2.084	0.000	Rejected
P19. House ownership	0.820	1.075	−3.153	4.024	0.030	Rejected
P20. Subjective wealth status	2.819	2.993	−4.321	11.632	0.374	Tentative

**Table 4:** Factors’ importance scores derived from Boruta algorithm for wasting status.

Code Variables	Mean Imp.	Median Imp.	Min. Imp.	Max. Imp.	Norm Hits	Decision
P1. SES	6.697	6.900	0.396	10.973	0.919	Confirmed
P3. MNK	−0.335	−0.504	−1.859	2.062	0.000	Rejected
P3. Stunting	18.184	18.585	9.927	26.268	1.000	Confirmed
P5. Underweight	57.893	59.973	41.257	68.979	1.000	Confirmed
P6. Child gender	−0.149	−0.605	−2.343	2.689	0.000	Rejected
P7. Health insurance	0.476	0.419	−1.494	2.018	0.000	Rejected
P8. Mother’s age	6.144	5.829	0.292	11.605	0.889	Confirmed
P9. Family size	1.704	1.821	−2.435	5.220	0.131	Rejected
P10. Under-five siblings	−1.388	−1.436	−2.333	−0.468	0.000	Rejected
P11. Exclusive BF	0.374	0.313	−2.203	2.160	0.000	Rejected
P12. Ever BF	−0.722	−0.460	−2.129	0.352	0.000	Rejected
P13. Initiation BF	1.491	1.604	−1.751	3.483	0.030	Rejected
P14. Weaning practices	1.546	1.626	−0.469	3.599	0.040	Rejected
P15. Mother education	3.604	3.399	−2.304	9.878	0.545	Confirmed
P16. Mother employment	0.775	0.370	−1.026	3.781	0.020	Rejected
P17. Family income	2.509	2.552	−2.512	6.517	0.465	Tentative
P18. Car ownership	1.872	1.729	−0.770	5.997	0.081	Rejected
P19. House ownership	4.088	3.901	−0.468	11.262	0.566	Tentative
P20. Subjective wealth status	−0.125	−0.535	−1.265	2.428	0.000	Rejected

**Table 5:** Factors’ importance scores derived from Boruta algorithm for underweight status.

Code Variables	Mean Imp.	Median Imp.	Min. Imp.	Max. Imp.	Norm Hits	Decision
P1. SES	1.184	1.103	−0.395	3.958	0.020	Rejected
P2. MNK	0.024	−0.150	−3.965	2.393	0.010	Rejected
P3. Stunting	40.846	42.037	28.338	48.492	1.000	Confirmed
P4. Wasting	65.480	67.689	44.840	74.953	1.000	Confirmed
P6. Child gender	0.033	0.158	−1.773	1.959	0.000	Rejected
P7. Health insurance	−1.061	−0.986	−2.302	0.035	0.000	Rejected
P8. Mother’s age	3.003	3.227	−2.405	9.000	0.444	Tentative
P9. Family size	1.713	1.838	−1.642	5.277	0.131	Rejected
P10. Under-five siblings	−0.146	−0.225	−1.900	1.331	0.000	Rejected
P11. Exclusive BF	3.086	2.995	−1.604	8.378	0.465	Tentative
P12. Ever BF	−0.969	−1.004	−2.206	0.412	0.000	Rejected
P13. Initiation BF	0.165	0.403	−2.381	2.601	0.010	Rejected
P14. Weaning practices	7.546	7.621	0.878	19.141	0.869	Confirmed
P15. Mother education	0.785	0.952	−1.683	2.981	0.010	Rejected
P16. Mother employment	1.233	1.422	−1.576	2.680	0.010	Rejected
P17. Family income	1.032	1.137	−2.124	3.358	0.010	Rejected
P18. Car ownership	0.085	0.109	−1.864	1.676	0.000	Rejected
P19. House ownership	−0.783	−0.622	−2.573	0.187	0.000	Rejected
P20. Subjective wealth status	3.578	3.490	−1.337	10.594	0.465	Tentative

## Data Availability

The raw data of this study are available upon request to the authors for the purpose of academic research. The data are not publicly available due to ethical and privacy restrictions, as they contain information that could compromise the confidentiality of study participants.

## Supplementary Materials

The following supporting information can be downloaded at https://www.mdpi.com/article/10.3390/nu17213355/s1, Table S1: Nutritional status of children aged 36–59 months in Surabaya, Indonesia.

## Abbreviations

The following abbreviations are used in this manuscript:

MNK	Maternal nutrition knowledge
CSDH	Conceptual Framework for Action on the Social Factors of Health
ICT	Information and Communication Technology
WebApps	Web applications
APK	Android Package Kit
BF	Breastfeeding
WHZ	Weight-for-height Z score
HAZ	Height-for-age Z score
WAZ	Weight-for-age Z score
WHO	World Health Organization
SES	Socioeconomic status
IYCF	Infant and young child feeding
GNKQ-R	General Nutrition Knowledge Questionnaire—Revised

## References

[1] UNICEF, WHO, World Bank (2023). Level and Trend in Child Malnutrition.

[2] Rahut D.B., Mishra R., Bera S. (2024). Geospatial and environmental determinants of stunting, wasting, and underweight: Empirical evidence from rural South and Southeast Asia. Nutrition.

[3] Maalouf-Manasseh Z., Oot L., Sethuraman K. Food and Nutrition Technical Assistance III Project Giving Children the Best Start in Life: Integrating Nutrition and Early Childhood Development Programming Within the First 1000 Days. 2016. https://www.fantaproject.org/sites/default/files/resources/Nutrition-Early-Childhood-Development-Technical-Brief-Jan2016.pdf.

[4] WHO, UNICEF WHO Child Growth Standards and the Identification of Severe Acute Malnutrition in Infants and Children: A Joint Statement by the World Health Organization and the United Nations Children’s Fund. 2009. p. 11. http://apps.who.int/iris/bitstream/10665/44129/1/9789241598163_eng.pdf.

[5] Briend A., Khara T., Dolan C. (2015). Wasting and stunting—Similarities and differences: Policy and programmatic implications. Food Nutr. Bull..

[6] Underweight Among Children Under 5 Years of Age (Number in Thousands) (Model-Based Estimates). https://www.who.int/data/gho/data/indicators/indicator-details/GHO/gho-jme-underweight-numbers-(in-millions).

[7] Siddiqui F., Salam R.A., Lassi Z.S., Das J.K. (2020). The Intertwined Relationship Between Malnutrition and Poverty. Front. Public Health.

[8] Lestari T.R.P. Stunting in Indonesia: Understanding the Roots of the Problem and Solutions. July 2023. https://berkas.dpr.go.id/pusaka/files/info_singkat/Info%20Singkat-XV-14-II-P3DI-Juli-2023-196-EN.pdf.

[9] Patel A.B., Kuhite P.N., Alam A., Pusdekar Y., Puranik A., Khan S.S., Kelly P., Muthayya S., Laba T., Almeida M.D. (2019). M-SAKHI—Mobile health solutions to help community providers promote maternal and infant nutrition and health using a community-based cluster randomized controlled trial in rural India: A study protocol. Matern. Child Nutr..

[10] Mistry S.K., Hossain B., Arora A. (2019). Maternal nutrition counselling is associated with reduced stunting prevalence and improved feeding practices in early childhood: A post-program comparison study. Nutr. J..

[11] Setia A., Shagti I., Boro R.A.M., Adi A.M., Saleh A., Sanjiwany P.A. (2020). The Effect of Family-Based Nutrition Education on the Intention of Changes in Knowledge, Attitude, Behavior of Pregnant Women and Mothers with Toddlers in Preventing Stunting in Puskesmas Batakte, Kupang Regency, East Nusa Tenggara, Indonesia Working Area. People.

[12] Black M.M., Delichatsios H.K., Story M.T. (2019). Nutrition Education: Strategies for Improving Nutrition and Healthy Eating in Individuals and Communities.

[13] Prasetyo Y.B., Permatasari P., Susanti H.D. (2023). The effect of mothers’ nutritional education and knowledge on children’s nutritional status: A systematic review. Int. J. Child Care Educ. Policy.

[14] Demilew Y.M., Alene G.D., Belachew T. (2020). Effect of guided counseling on nutritional status of pregnant women in West Gojjam zone, Ethiopia: A cluster-randomized controlled trial. Nutr. J..

[15] Wahyurin I.S., Aqmarina A.N., Rahmah H.A., Hasanah A.U., Silaen C.N.B. (2019). Effect of stunting education using brainstorming and audiovisual methods towards knowledge of mothers with stunted children. Ilmu Gizi Indones..

[16] Suryati S., Supriyadi S. The Effect of booklet education about children nutrition needs toward knowledge of mother with stunting children in Pundong primary health center work area Bantul Yogyakarta. Proceedings of the 4th International Nursing Conference.

[17] Dinengsih S., Hakim N. (2020). The influence of the lecture method and the android-based application method on adolescent reproductive health knowledge. J. Kebidanan Malahayati.

[18] Kassaw M.W., Bitew A.A., Gebremariam A.D., Fentahun N., Açık M., Ayele T.A. (2020). Low Economic Class Might Predispose Children under Five Years of Age to Stunting in Ethiopia: Updates of Systematic Review and Meta-Analysis. J. Nutr. Metab..

[19] Nassanga P., Okello-Uma I., Ongeng D. (2018). The status of nutritional knowledge, attitude and practices associated with complementary feeding in a post-conflict development phase setting: The case of Acholi sub-region of Uganda. Food Sci. Nutr..

[20] Ekawidyani K.R., Khomsan A., Dewi M., Thariqi Y.A. (2022). Nutrition Knowledge, Breastfeeding and Infant Feeding Practice of Mothers in Cirebon Regency. Amerta Nutr..

[21] Jusoh N., Lee J.L.F., Tengah R.Y., Azmi S.H., Suherman A. (2021). Association between nutrition knowledge and nutrition practice among Malaysian adolescent handball athletes. Malays. J. Nutr..

[22] Bukusuba J., Kikafunda J.K., Whitehead R.G. (2010). Nutritional Knowledge, Attitudes, and Practices of Women Living with HIV in Eastern Uganda. J. Health Popul. Nutr..

[23] A Conceptual Framework for Action on the Social Determinants of Health. https://www.who.int/publications/i/item/9789241500852.

[24] National Academies of Sciences Frameworks for Addressing the Social Determinants of Health. October 2016. https://www.ncbi.nlm.nih.gov/books/NBK395979/.

[25] Alristina A.D., Mahrouseh N., Irawan A.S., Laili R.D., Zimonyi-Bakó A.V., Feith H.J. (2025). Prematurity and Low Birth Weight Among Food-Secure and Food-Insecure Households: A Comparative Study in Surabaya, Indonesia. Nutrients.

[26] Marrie R.A. (2011). Demographic, Genetic, and Environmental Factors That Modify Disease Course. Neurol. Clin..

[27] Socioeconomic Status. https://www.apa.org/topics/socioeconomic-status.

[28] McKinsey & Company Personalized Medicine—The Path Forward. Translational Informatics. 2013. pp. 35–60. https://www.mckinsey.com/industries/life-sciences/our-insights/mckinsey-personalized-medicine-compendium-the-path-forward.

[29] Fiala M.A., Finney J.D., Liu J., Stockerl-Goldstein K.E., Tomasson M.H., Vij R., Wildes T.M. (2015). Socioeconomic Status is Independently Associated with Overall Survival in Patients with Multiple Myeloma. Leuk. Lymphoma.

[30] Gao L., Sun B., Du Z., Lv G. (2022). How Wealth Inequality Affects Happiness: The Perspective of Social Comparison. Front. Psychol..

[31] Australian Centre on Quality of Life. https://www.acqol.com.au/instruments.

[32] Miller B.K., Zivnuska S., Kacmar K.M. (2019). Self-perception and life satisfaction. Pers. Individ. Differ..

[33] WHO, UNICEF (2021). Indicators for Assessing Infant and Young Child Feeding Practices: Definitions and Measurement Methods.

[34] Kliemann N., Wardle J., Johnson F., Croker H. (2016). Reliability and validity of a revised version of the General Nutrition Knowledge Questionnaire. Eur. J. Clin. Nutr..

[35] Yanagihara Y., Narumi-Hyakutake A. (2025). Relationship between nutrition knowledge and nutritional adequacy in Japanese university students: A cross-sectional study. J. Nutr. Sci..

[36] Saleem J., Zakar R., Butt M.S., Aadil R.M., Ali Z., Bukhari G.M.J., Ishaq M., Fischer F. (2024). Application of the Boruta algorithm to assess the multidimensional determinants of malnutrition among children under five years living in southern Punjab, Pakistan. BMC Public Health.

[37] Kursa M.B., Rudnicki W.R. (2010). Feature Selection with the Boruta Package. J. Stat. Softw..

[38] Jaringan Dokumentasi dan Informasi Hukum. https://dokumjdih.jatimprov.go.id/arsip/info/48965.html.

[39] Pandey S. (2013). Socio-economic and Demographic Determinants of Antenatal Care Services Utilization in Central Nepal. Int. J. Matern. Child Health AIDS.

[40] Sánchez T.E., Linehan L., O’Donoghue K., Byrne M., Meaney S. (2022). Facilitators and barriers to seeking and engaging with antenatal care in high-income countries: A meta-synthesis of qualitative research. Health Soc. Care Community.

[41] Yadav A.K., Sahni B., Jena P.K. (2020). Education, employment, economic status and empowerment: Implications for maternal health care services utilization in India. J. Public Aff..

[42] Tahreem A., Rakha A., Anwar R., Rabail R., Maerescu C.M., Socol C.T., Criste F.L., Abdi G., Aadil R.M. (2025). Impact of maternal nutritional literacy and feeding practices on the growth outcomes of children (6–23 months) in Gujranwala: A cross-sectional study. Front. Nutr..

[43] Harvey C.M., Newell M.-L., Padmadas S. (2022). Maternal socioeconomic status and infant feeding practices underlying pathways to child stunting in Cambodia: Structural path analysis using cross-sectional population data. BMJ Open.

[44] Gbratto-Dobe S.A., Segnon H.B. (2025). Is mother’s education essential to improving the nutritional status of children under five in Côte d′Ivoire?. SSM—Health Syst..

[45] Phyo W.Y., Khin O.K., Aung M.H. (2021). Mothers’ Nutritional Knowledge, Self-efficacy, and Practice of Meal Preparation for School-age Children in Yangon, Myanmar. Makara J. Health Res..

[46] Thomas D., Strauss J., Henriques M.-H. (1991). How Does Mother’s Education Affect Child Height?. J. Hum. Resour..

[47] Prickett K.C., Augustine J.M. (2015). Maternal Education and Investments in Children’s Health. J. Marriage Fam..

[48] Wang W.-C., Zou S.-M., Ding Z., Fang J.-Y. (2023). Nutritional knowledge, attitude and practices among pregnant females in 2020 Shenzhen China: A cross-sectional study. Prev. Med. Rep..

[49] Forh G., Apprey C., Agyapong N.A.F. (2022). Nutritional knowledge and practices of mothers/caregivers and its impact on the nutritional status of children 6–59 months in Sefwi Wiawso Municipality, Western-North Region, Ghana. Heliyon.

[50] Shekutamba A.F., Ashipala D.O. (2023). Nutritional knowledge and practices of mothers with malnourished children in a regional hospital in Northeast Namibia. J. Public Health Afr..

[51] Atsu B.K., Guure C., Laar A.K. (2017). Determinants of overweight with concurrent stunting among Ghanaian children. BMC Pediatr..

[52] Chowdhury M.R.K., Rahman S., Billah B., Kabir R., Perera N.K.P., Kader M. (2022). The prevalence and socio-demographic risk factors of coexistence of stunting, wasting, and underweight among children under five years in Bangladesh: A cross-sectional study. BMC Nutr..

[53] Soekatri M.Y.E., Sandjaja S., Syauqy A. (2020). Stunting Was Associated with Reported Morbidity, Parental Education and Socioeconomic Status in 0.5–12-Year-Old Indonesian Children. Int. J. Environ. Res. Public Health.

[54] Silas V.D., Pomat W., Jorry R., Emori R., Maraga S., Kue L., Berry N., Aga T., Luu H.N., Ha T.H. (2023). Household food insecurity during the COVID-19 pandemic and associated socioeconomic demographic factors in Papua New Guinea: Evidence from the Comprehensive Health and Epidemiological Surveillance System. BMJ Glob. Health.

[55] Arini D., Ernawati D., Hayudanti D., Alristina A.D. (2022). Impact of socioeconomic change and hygiene sanitation during pandemic COVID-19 towards stunting. Int. J. Public Health Sci. (IJPHS).

[56] Emmanuel N.B., Mala Ali M., Celestin B.L.N., Kalombola C., Gérard M.M., Bavon T.M., Patient N.B., Crédo K.T., Déogratias M.N.H., Oscar L.N. (2023). Complementary Feeding Practices Associated with Malnutrition in Children Aged 6–23 Months in the Tshamilemba Health Zone, Haut-Katanga, DRC, 2021. Acta Sci. Nutr. Health.

[57] Thuita F., Mukuria A., Muhomah T., Locklear K., Grounds S., Martin S.L. (2021). Fathers and grandmothers experiences participating in nutrition peer dialogue groups in Vihiga County, Kenya. Matern. Child Nutr..

[58] Martin S.L., McCann J.K., Gascoigne E., Allotey D., Fundira D., Dickin K.L. (2021). Engaging family members in maternal, infant and young child nutrition activities in low- and middle-income countries: A systematic scoping review. Matern. Child Nutr..

[59] Adhikari N., Acharya K., Upadhya D.P., Pathak S., Pokharel S., Pradhan P.M.S. (2021). Infant and young child feeding practices and its associated factors among mothers of under two years children in a western hilly region of Nepal. PLoS ONE.

[60] Ijaiya M.A., Anjorin S., Uthman O.A. (2024). Income and education disparities in childhood malnutrition: A multi-country decomposition analysis. BMC Public Health.

[61] Morales F., la Paz S.M.-D., Leon M.J., Rivero-Pino F. (2023). Effects of Malnutrition on the Immune System and Infection and the Role of Nutritional Strategies Regarding Improvements in Children’s Health Status: A Literature Review. Nutrients.

[62] Noort M.W.J., Renzetti S., Linderhof V., du Rand G.E., Marx-Pienaar N.J.M.M., de Kock H.L., Magano N., Taylor J.R.N. (2022). Towards Sustainable Shifts to Healthy Diets and Food Security in Sub-Saharan Africa with Climate-Resilient Crops in Bread-Type Products: A Food System Analysis. Foods.

[63] Ogunniran O.P., Ayeni K.I., Shokunbi O.S., Krska R., Ezekiel C.N. (2024). A 10-year (2014–2023) review of complementary food development in sub-Saharan Africa and the impact on child health. Compr. Rev. Food Sci. Food Saf..

[64] Oniang’o R., Maingi Z., Jaika S., Konyole S. (2025). Africa’s contribution to global sustainable and healthy diets: A scoping review. Front. Nutr..

[65] Asebe H.A., Asmare Z.A., Mare K.U., Kase B.F., Tebeje T.M., Asgedom Y.S., Shibeshi A.H., Lombebo A.A., Sabo K.G., Fente B.M. (2024). The level of wasting and associated factors among children aged 6–59 months in sub-Saharan African countries: Multilevel ordinal logistic regression analysis. Front. Nutr..

[66] Headey D.D., Ruel M.T. (2022). Economic shocks predict increases in child wasting prevalence. Nat. Commun..

[67] Lawal S.A., Okunlola D.A., Adegboye O.A., Adedeji I.A. (2024). Mother’s education and nutritional status as correlates of child stunting, wasting, underweight, and overweight in Nigeria: Evidence from 2018 Demographic and Health Survey. Nutr. Health.

[68] Okutse A.O., Athiany H. (2025). Socioeconomic disparities in child malnutrition: Trends, determinants, and policy implications from the Kenya demographic and health survey (2014–2022). BMC Public Health.

[69] Ajmal S., Ajmal L., Ajmal M., Nawaz G. (2022). Association of Malnutrition with Weaning Practices Among Infants in Pakistan. Cureus.

[70] Pereira T.A.d.M., Freire A.K.G., Gonçalves V.S.S. (2020). Exclusive breastfeeding and underweight in children under six months old monitored in primary health care in Brazil, 2017. Rev. Paul. Pediatr..

[71] Erda R., Hamidi D., Desmawati D., Rasyid R., Sarfika R. (2025). Evaluating socio-demographic, behavioral, and maternal factors in the dual burden of malnutrition among school-aged children in Batam, Indonesia. Narra J.

[72] Roswita N., Dartanto T. (2024). Maternal Decision Making and Children’s Nutritional Status: Evidence from Indonesia. J. Èkon. Kependud. Kel..

[73] Dembedza V.P., Mapara J., Chopera P., Macheka L. (2025). Relationship between cultural food taboos and maternal and child nutrition: A systematic literature review. N. Afr. J. Food Nutr. Res..

[74] Lekey A., Masumo R.M., Jumbe T., Ezekiel M., Daudi Z., Mchome N.J., David G., Onesmo W., Leyna G.H. (2024). Food taboos and preferences among adolescent girls, pregnant women, breastfeeding mothers, and children aged 6–23 months in Mainland Tanzania: A qualitative study. PLoS Glob. Public Health.

[75] Kayumba R. (2023). To Explore the Perceived Food Taboos during Pregnancy and their Relation to Maternal Nutrition and Health. TEXILA Int. J. Acad. Res..

[76] UNICEF (1990). Strategy for Improved Nutrition of Children and Women in Developing Countries.

[77] ECDAN Child Nutrition Report 2025. https://ecdan.org/news/child-nutrition-report-2025/.

