# Supplementary material for: The Importance of Socioeconomic Factors Associated with Maternal Nutrition Knowledge and Undernutrition Among Children Under Five
Source: Nutrients. 2025 Oct 24;17(21):3355. doi: 10.3390/nu17213355 (PMC12610531; doi:10.3390/nu17213355)
Supplement: Supplementary file 1 [file nutrients-17-03355-s001.zip › nutrients-3906561-supplementary.pdf]

Table S1 Nutritional status of children aged 36-59 months in Surabaya, Indonesia

**Underweight n (%)**	**Stunting n (%)**	**Wasting n (%)**		**Total**
		Yes	No	
Yes	Yes	24 (16.2)	58 (39.2)	82 (55.4)
	No	52 (35.1)	14 (19.5)	66 (44.6)
Total		76 (51.4)	72 (48.6)	148 (100.0)
No	Yes	0 (0.0)	84 (16.5)	84 (16.5)
	No	30 (5.9)	395 (77.6)	425 (83.5)
Total		30 (5.9)	479 (94.1)	509 (100.0)
Total Stunting	Yes	24 (3.7)	142 (21.6)	166 (25.3)
	No	82 (12.5)	409 (62.3)	491 (74.7)
Total		106 (16.1)	551 (83.9)	657 (100.0)
